# The Pathology of Asthma1Read before the Section on Pathology, Buffalo Academy of Medicine, April 16, 1901.

**Published:** 1901-06

**Authors:** G. N. Jack

**Affiliations:** Depew, N. Y.


### THE PATHOLOGY OF ASTHMA.[1]

By G. N. JACK, M. D., Depew, N. Y.

IN CONSTRUCTING a pathology for asthma I find it no easy task, for as far as I can ascertain this is the first attempt at venturing so far in the realms of this heretofore mysteriously considered disease. In the spirit of the present age the knowledge of no disease is considered perfect without a well defined pathology, obtained in most cases by a careful post mortem study of the diseased tissues. But the pathology of asthma could never have been discovered nor proven, neither can much light be thrown upon it by post mortem investigations. The pathology of asthma being an abnormal biochemical process, is therefore necessarily destroyed and masked by death. But with our modern, and reasonably accurate methods of studying living tissues and life in its complexity, it is safe and due time to venture the penetration of that halo of mystery hovering around biochemical diseases.

The pathology of asthma, then, is to be learned and studied mostly, and, in many cases, entirely from the living subject. No observer, no matter how acute, could ever hope to discover the pathology of asthma, without first knowing its etiology, symptoms and cure. In other words, the key to the door of the pathological laboratory of asthma was discovered by the light of its etiology, and this etiology was determined largely by a symptomatic and curative process of reasoning. Having determined the etiology, symptoms and cure, the pathology works itself out with a mathemtical exactness which none can dispute nor deny. By this process of reasoning we would have the following algebraic equasion:

1. Read before the Section on Pathology, Buffalo Academy of Medicine, April 16, 1901.

Given the etiology, symptoms and cure to find the pathology, we have:

| Etiology. | Symptoms. | Cure. | Pathology. |
|---|---|---|---|
| Intestinal indigestion and toxemia with its resultant abnormalities of the fluids of the body, especially the blood lymph and chyle. | Dyspnea. | Two and one-half to three years' treatment by a fresh animal and green vegetable diet, with stools and blood as a guide, exercise, cold - water baths, habits, etc. | X |

It will be seen, then, that asthma is not a disease by itself having a well-established entity, but that it is only a symptom, a part of a vicious circle, or an abnormal biochemical and complex pathological process, originating usually in the intestinal canal, through a long-standing intestinal indigestion and toxemia, with faulty absorption and metabolism, producing a toxic or lymphagenous chyle that generates an unstable blood, characterised by its extremely varied, numerous and alarming paroxysmal, morphologic changes, often alternating between a lymphocytosis, an intestinal toxemic leucocytosis, or a marked anemia; accompanied anatomically by a hyperplasia of the lymphatic and glandular structures and clinically by a most wretched and agonising dyspnea. This definition makes it plain that the one characteristic pathological feature of asthma is the unstableness of its blood.

During long periods of quiesence the blood of the asthmatic usually presents no demonstrable pathognomonic constituents, but repeated blood analyses during attacks most vividly picture its unstableness. During some attacks the blood will present quite constant pathologic changes, indicating throughout the attacks a decided lymphocytosis, an intestinal toxemic leucocytosis or an anemia, but most frequently we find the blood rapidly oscillating between these three varieties, and occasionally there will be a blending of two of the three abnormalities or of the entire group. In previous papers on this subject read before this Academy, and published in the Buffalo Medical Journal, February, 1899, and January, 1900, I gave approximately a similar classification of blood abnormalities as the etiology of asthma, although in those classifications I was inclined to believe that that which I have now discovered to be an intestinal toxemic leucocytosis was more of a lymphatic leukemia.

In regard to one of these papers Osler wrote me the following kind and encouraging letter:

"BALTIMORE, January 26, 1900.

"DEAR DR. JACK:

"I was much interested in your paper on the Hygiene of the asthmatic, in which I found many good points. I have never had my attention called to any special relation between leukemia and asthma, and I don't know that the eosinophilia, which is so constant a feature, has any special suggestion in this respect.

Sincerely yours, WILLIAM OSLER."

I should infer from this note that my present classification will still more agreeably accord with Osler's views. Owing to the fact that the three varieties of asthma herein considered so frequently blend, or rapidly alternate, they can hardly be classed as separate pathological conditions, yet as it would be rather confusing to undertake to construct a combined pathology applicable to these manifestations, it is deemed best here to deal with them singly. The nomenclature of the group is taken from the blood dyscrasia most prominent in the production of the asthmatic dyspnea and accordingly are (1) a lymphocytosis; (2) an intestinal toxemic leucocytosis; (3) an anemia.

I. *Asthmatic Lymphocytosis.*—The pathology of this form of asthma lies chiefly in the blood, and in uncomplicated cases there usually is no other appreciable lesion. The asthmatic lymphocytosis is most frequently found in (*a*) children during lactation; (*b*) in asthmatic adults subsisting largely upon a lymphogenous diet.

(*a*) Children of an asthmatic tendency are very apt, especially during lactation, to suddenly develop a lymphocystosis that results in a serious, grave, and often fatal, dyspnea. The attacks usually come on suddenly in the latter part of the night without any prodromal symptoms or exciting causes, other than the lack of the chemical effect of sunlight on the blood. The child may be thrifty, well-nourished, fleshy and rapidly developing, but, as a rule, it is flabby muscled, more or less rhachitic and peevish.

This peevish, irritable, worrisome and changeable disposition, especially pronounced just preceding or during short intervals of attacks, is quite characteristic and offers an excellent clinical demonstration of the unstableness of the blood of the asthmatic. The kidney is the first organ to detect this lymphogenous change in the blood and when this change is gradual, and the kidney normal, it will filter off the excess of lymph almost as fast as it forms, which renders the attacks very mild. The mother will usually observe that the child wets an extraordinary large number of diapers a day or so before an attack. The urine is of a light color, low specific gravity, odorless, neutral in reaction and contains but few solids or crystalline substances. This diuresis diminishes during an attack, to become quite scanty, high colored and highly acid with its cessation. If, with this diuresis a diarrhea mercifully sets in, the excess of lymph will often be drained off sufficiently to completely avert the attack.

The blood analyses are characteristically variable, but the majority will indicate a relative increase in the lymphocytes. Owing to the dilution of the blood by this excessive lymph formation, there is a reduction in the percentage of the hemoglobin, as denoted by the quite constantly lowered specific gravity of the blood. The eosinophiles are decidedly increased in numbers, but still no more so than one would expect in a child, from the malnutrition resulting from a disturbed or perverted metabolism sufficient to produce the lymphocytosis. The most probable cause of this temporary malnutrition and perverted metabolism during lactation is that, from some slight exciting cause, the digestive ferments do not properly act upon the ingested milk which permits the formation of a lymphogenous chyle that, when absorbed by the lacteals and carried to the blood, acts more as a lymphogogue than a true hematogenic substance. The two cardinal factors in the production of the asthmatic dyspnea when due to a lymphocytosis are (1) the hemoglobin cannot reach the oxygen in the lungs; (2) the oxygen cannot reach the lungs.

The hemoglobin is handicapped in reaching the oxygen in the lungs, first, by its being diluted with and thickly surrounded by lymphocytes; and, second by a thickened and air-tight condition of the partition between the oxygen and hemoglobin, due to an accumulation of mucus in the lung substance. The hemoglobin is also often in grave or fatal cases still further excluded from oxygen, by a collateral engorgement of the lung capillaries with lymph. The oxygen is debarred from reaching the lungs by their partially filling with mucus together with the trachea and larynx. In favorable cases, after an hour or so, the pathology changes and the lymph is gotten rid of, by some being metamorphosed into normal blood substance, and the rest by elimination.

A recent case that shows the serious aspect that this variety of asthma is apt to assume, and also serves to clench its pathology, was that of a breast-fed, well nourished, healthy parented, Polish asthmatic child of ten months. It was never entirely free from asthma, but occasionally the attacks would become alarming. In one of these severe paroxysms the respiratory apparatus was so completely filled with mucus, that the most labored respiratory efforts would only allow a discouragingly small amount of air to enter. It is needless to note that this condition was accompanied by cyanosis, lividity, and agonising appeals. Relief, however, was soon obtained by draining the lymph from the blood, and expelling the mucus from the respiratory apparatus. Such heroic measures were resorted to as the administration of a hot saturated solution of epsom salts per stomach until a free emesis was established, a high rectal irrigation with the same solution and followed by holding the child up by the heels, shaking, spanking and tapping on the chest,to aid the expulsion of mucus from the respiratory apparatus.

The blood count showed 93,500 white cells, 70 per cent. of which were lymphocytes. A vast majority of these lymphocytes were small, showing that they had come directly from the lymph channels and the chyle. The child rapidly rallied after this attack, but milk was not allowed for several days on account of its lymphogenous nature, but was substituted by pure blood in the form of bovinine and panopeptone. For the succeeding four weeks the child seemed to thrive and do remarkably well, having only a few milds attacks of asthma. At this period it had another severe attack, like the one above described, in which it died, having no medical assistance, as I arrived only in time to see it expire. A careful post mortem was held, but nothing pathological gained.

The average asthmatic nursling, however, usually presents a somewhat different pathological picture. As a rule, there is a less marked lymphocytosis and more anemia. The lymphocytosis frequently results in adenoids of the nasopharynx, adenolymphoceles, and more or less engorgement and hypertrophy of the lymph spaces of the entire respiratory tract.

(*b*) The asthmatic lymphocytosis of adults, is only seen in asthma of long standing, where the blood has a strong tendency to return to the infantile type, and in cases that have been kept too exclusively upon a lymphogenous diet, as milk, raw eggs, or raw oysters. The lymphocytosis is also induced or augmented by any agent that tends to impair the nutritive value of the plasma. The pathology when occurring in adults does not differ much from that of the infant as above described.

II. *Asthma due to an Intestinal Toxemic Leucocytosis.*—This is by far the most important group. Its pathology begins with the intestinal indigestion and toxemia, which usually continually exists in a variable degree, having, as a rule, a marked exacerbation preceding an attack. This exacerbation in the intestinal toxemia is especially pronounced when preceding an attack following a comparatively long interval of freedom. After the vicious circle of the asthmatic has become established, and the attacks continue or follow at short intervals, the process becomes chronic, and there are only slight exacerbations in the intestinal toxemia.

A case under my present observation has most obviously pictured this vicious circle, and proved its importance in the pathology of asthma.

Male, aged 31; had asthma from infancy to age 10. No asthma from age 10 to age 22. Asthma from age 22 to present. Present attack began last October, after five or six months freedom. All last summer had intestinal indigestion, as manifested by a constant alteration between watery mucus, liquid, fetid, or hard,dry stools, flatulency, and urinary disturbance.

About three weeks before the attack,toxic products were absorbed from the intestinal canal sufficient to produce an enlargement of the numerous glands situated in the larynx and trachea. This glandular enlargement could be detected by the snugness of the collar, and change of voice. The vocal chords also had difficulty in adapting themselves in speech, and would soon become exhausted. The increase in the size of the neck previous to and during the attack was so pronounced that patient was compelled to keep two sets of collars on hand, one size one inch and a half larger than the normal for the asthmatic period.

The blood's faithful guardian, the kidney, was the next organ to become irritated and worried over the blood's dangerous situation, and endeavored to correct matters by the filtering off of as much as possible of the waste material from the already rapidly disintegrating blood. Indican could occasionally be detected, but about the only catobolic evidence in the urine at this stage was its paleness and its excessiveness.

The urine of the asthmatic is as changeable as the unstable blood from which it is filtered. At one urination it may be pale, abundant and of a very low specific gravity, to become in a few hours, especially if following an attack, scanty, high colored, from a disintegration of blood substance, and loaded with uric acid from an increased destruction of leucocytes. The characteristically high acidity of the urine during and following attacks, furnishes another evidence of the blood's diminished alkalinity during attacks. From seven to ten days previous to the attack, the intestinal condition became decidedly toxic; the stools were small, frequent, soft, accompanied by much flatus, and possessed of a nauseating, disgusting, irritating and penetrating odor, that would force the inspector to turn away.

After such an inspection one does not wonder that the blood, a substance essentially sensitive to gases, should rapidly undergo a disintegration from having the field from which it absorbs its sustenance, as the intestinal canal, encompassing such a gaseous toxic material. The stools would change in character from an acid, gaseous, fermenting and irritating condition to an alkaline, putrid, cadaveric and mucous one. This intestinal toxemic condition continued, and during the asthmatic attack with days of improvement, to be followed by an exacerbation.

At the stage of intestinal toxemia, the urine began to show decided evidences of a destructive metamorphosis. It became scanty, exceedingly high colored, of a high specific gravity, and loaded with uric acid. The indicanuria was decidedly augmented.

About twenty-four hours before the attack patient had a severe chill, lasting half an hour; the chill had no febrile reaction, and on the following day the patient felt as well as usual. In the evening, however, his voice had a heavy, congested, bass-like sound, and the collar seemed unusually tight.

For the five months prior to the attack, during which time the patient had no dyspnea, his blood was analysed nearly every third day, owing to the fact that he had been put almost exclusively upon a certain diet (the details of which cannot here be considered) in order to test its efficiency in the treatment of asthma. During most of these blood analyses the patient was in fairly good health and nothing pathological could be demonstrated. Yet, not infrequently, one could detect an appreciable increase in the leucocytes and eosinophiles.

The unstable nature of the blood was often manifested by its tendency to go to extremes in many physiological processes. Thus, the normal leucocytosis of digestion would at times be so greatly exaggerated as to become, for a short time, pathological. The unstableness and extremism of the blood was manifested clinically in the patient by his becoming unnaturally and almost uncontrollably exhilarated under the beneficial influence of sunshine, altitude, or after a cold sponge bath, which was followed by an extreme degree of melancholy under the blood debilitating influence of night darkness, damp, cloudy days, or wet, muddy, swampy districts. The normal drowsiness accompanying the digestion leucocytosis would occasionally be so exaggerated as to require a strong wilful effort to maintain wakefulness. The unstableness of the blood was, of course, directly due to its poorly nourished condition resulting from the intestinal toxemia.

During the stage accompanied by the acid, fermenting, stools, there was a perceptible diminution in the alkalinity of the blood. After the chill the blood began to show pronounced pathological features. The leucocytic count rapidly rose to 61,000, 30 per cent. of which were eosinophiles. After this blood count the on-coming asthmatic attack was positively foretold. This count would rapidly oscillate throughout the attack, but there was constantly and decidedly a pathological leucocytosis.

While it is as yet an undecided question as to the relation that the eosinophiles have to the other white blood corpuscles or their exact significance when occurring in either normal or abnormal numbers in the peripheral blood, nevertheless, occurring, as they do, in such excessive numbers in a condition like this, where the mortality of the leucocytes greatly exceeds their nativity, owing to a toxic and illy nourished condition of the blood, the said toxemia originating from one of the breeding places of the eosinophiles or the intestinal canal, would forcibly indicate that the leucocytes have an eosinophilic origin. This hypothesis would afford two rational explanations for their appearing in such excessive numbers in the blood of the asthmatic. First, that they had migrated into the blood substance to hasten the formation of new leucocytes; and, second, that they had been forced to abandon their normal breeding places in the lymph tissue of the intestinal canal, from its toxic condition. Thus it will be seen, that all of the pathological constituents found in this variety of asthmatic blood are directly due to the rapid completion of the life cycle of the leucocyte, with its consequent pathological representation of all of its life cycle phases, as its origin, existence, death and remains, which would be respectively represented by the eosinophiles, leucocytes, uric acid and possibly Charcot-Leyden crystals.

The dead leucocytes would be represented by those that the blood so rapidly and abundantly casts off through the mucous membranes. During the last stages of the attack the red corpuscles began to be noticeably affected by the toxic plasma, as manifested by their slightly imperfect rouleaux formation.

The dyspnea came on gradually, as it frequently does in the toxic variety after comparatively long periods of freedom. The first respiratory obstruction appeared in the larynx from an enlargement of the glands there situated, the patient experiencing difficulty in expiring the air, and each expiration was accompanied by a loud whistling, wheezing noise, coming directly from the larynx.

In two hours' time there seemed to be almost a complete occlusion of the larynx and asphyxia seemed imminent. A glance at the anatomical structure of the larynx, will readily explain the mechanism of laryngeal dyspnea with its painfully prolonged difficult wheezing expirations.

The ventricular bands or false chords located in the larynx have a valve-like arrangement, said valve having its free edges extending downward in such a manner that the ingress of air would tend to open the valve, and its egress to close it. In normal respirations this valve has no action, but in the pathology of laryngeal dyspnea it plays a very important rôle. The ventricular bands are so thickened and hypertrophied by the accumulation of waste fluids from the rapidly disintegrating blood, in the numerous wide lymph spaces found in them, that they project into the lumen of the larynx far enough to catch sufficient air on expiration to produce a valvular action that nearly closes the air-tube. The process is also aggravated and the ventricular bands pushed farther into the lumen of the larynx by a great engorgement with dead leucocytes, of the many mucous glands located in the ventricles of the larynx and their ascending pouches.

The agony of the patient at this period surpasses description, and one can read the pathology in his very attitude. He stands erect to bring gravity to his aid in lessening the lymph space and glandular engorgement, while tightly grasping some solid object with his hands, to bring in action all of the auxiliary muscles possible to aid in forcing the air out of the lungs. The teeth are set and the sterno-cleido-mastoid muscles of the neck rigid, in their effort to hold the larynx in the most favorable position for the passage of air, but in spite of this attempt at fixation, the larynx is forced up with each expiration and down with each inspiration: the wheezing is confined to the region of the larynx. Each inspiration is begun with a voluntary snuff and jerk in order to lessen the respiratory pause. A voluntary groan is produced on expiration, which presumably helps to prevent the valvular action of the swollen ventricular bands. Phonation is almost impossible, and when forced to talk it seems to exhaust the last bit of air left in the lungs. After about twenty-four hours of that laryngeal dyspnea, the glands underwent a resolution and degeneration, with the expulsion of thick mucus, which was expectorated in lumps the size of a pigeon's egg and of a tough, tenacious, viscid consistency, which gave it an appearance of fat tissue and enabled one to pick it up and handle it about with the fingers.

On inspection it was found to contain several little grayish, pearly balls, which on being unravelled and viewed under the microscope were found to be composed of delicate convoluted spirals (Curschmann's), made up of numerous individual filaments. Other portions of the sputum under the microscope, without staining, were found to contain numerous leucocytes, exhibiting bright, yellowish, coarse granulations; among these were numerous colorless pointed octohedral crystals (Charcot-Leyden's). Disseminated throughout the field were numerous eosinophile granules derived from ruptured eosinophile cells.

Ever since the discovery of spirals in the sputum of the asthmatic by Curschmann it has been a mystery as to their origin, but most authorities believe them to come from the smallest bronchioles. This theory can now be proven erroneous by the fact that they are abundantly found in the sputum coming direct from the larynx, before the process has extended into the bronchioles at all. After the process has extended into the trachea and bronchioles, the sputum first exuded here, also from the involved lymphatic tissue, likewise contains these same spirals, but the fact that they are contained in the sputum coming direct from the larynx before the process has extended beyond that region, proves conclusively that the spirals do not in any case originate in the smallest bronchioles as their casts as heretofore supposed. Where, then, do they originate?

In follicular tonsilitis one often is able to squeeze from the ducts of the inflamed gland perfect casts of these ducts, sometimes one-eighth of an inch long. In laryngeal asthma all of the numerous glands, located in the larynx are engorged to their utmost capacity, with dead leucocytes and this engorgement is maintained, with an increasing pressure by the blood's continuing to dump dead leucocytes there. This pressure causes a plugging of the minute ducts of the small glands with dead leucocytes, until finally the tissue about the duct softens and gives way, which permits the expulsion of the filament-like plug. As these filament-like plugs individually exude from the numerous closely crowded minute ducts, into the lumen of the air-tube, their free ends are caught by the air as it passes out and in and twisted together, making the convoluted spirals. That this is the true origin of these spirals is still further proven by the fact that they are contained only in the mucus first expectorated, which would show that after the glands once softened, and their minute ducts dilated, they expel their contents as fast as formed, without the "duct plugging" or filament formation. The leucocytes, eosinophiles and octohedral crystals found in the sputum, do, of course, come directly from the leucocytic blood. After the softening of the laryngeal glands and the expectoration of their contents, the patient had a few hours of ease with normal respiration, when the dyspnea began again, due to an engorgement of the glands of the trachea and bronchioles. The air would now pass in and out through the larynx freely, and without any wheezing, to meet with obstructions farther down, as indicated by the location of the dry râles and the attitude of the patient. The air now meets with no more obstructions during expiration than it does during inspiration, in which respect it differs from the laryngeal dyspnea. The expirations are, however, here also somewhat prolonged owing wholly to the physiological mechanism of the respiratory muscles. Through the powerful, and if need be, voluntary contraction of the diaphragm an immediate inspiratory force can be produced great enough to rapidly fill the lungs, through a narrowed tube, with a volume of air that will require a painfully long time for the comparatively weak expiratory force to expel.

Owing to the blood's diminished alkalinity together with its leucocytic condition, it is unable to utilise but a small portion of the oxygen that required such a strained muscular effort to furnish, hence the blood reaches the respiratory center in the medulla in a poorly oxygenated condition, which irritates the pneumogastric nerve, thus continually urging the diaphragm on to a still more rapid and strained muscular action, regardless of the quantity of oxygen the lungs may already contain.

In the nonobstructed dyspnea this violent action of the diaphragm will not injure the lung tissue, as enough air will escape with each expiration before the hurried beginning of another inspiration, to prevent their excessive dilatation; but not so where the dyspnea is complicated by an obstruction in the air-tube, for then the normally passive and, when forced by all of its auxiliary muscles, the much weaker expiratory action, will not be permitted time enough, by the agitated diaphragm, to expel more than a part of the inhaled air before the beginning of another forced inspiration which tends to excessively dilate the lungs and produce a temporary emphysematous condition, that if long continued becomes more or less permanent.

The duration of this tubercular obstructed dyspnea was about the same as that of the laryngeal, and it disappeared by the same pathological process, throwing off the same thick, tenacious mucus which, however, seemed to be expectorated in smaller sized lumps. With the beginning of the expectoration a constant cough developed, eliminating in a few hours over a pint of thick, sticky mucus, loaded with the white balls containing the asthma spirals. So thick was this mucus that when its receptacle was jarred the mass would tremble like so much jelly, and when diluted with water and emptied, it would adhere to the sides of its retainer and string down for nearly two feet, and if the vessel were turned back slowly a large amount of the mucus would draw back into it. Mucus of this nature was expectorated for about ten hours, after which the spirals gradually disappeared; with the disappearance of the spirals the expectorations became more profuse; the quantity of mucus expectorated at this period was appalling.

The cough was constant, and each cough usually resulted in the raising of as large a quantity of leucocytes or mucus as could be forced into the mouth. While this pouring out of leucocytes into the air-tubes, with their free expectoration continued without interruption, there was but little if any dyspnea; but if for any reason the blood had its vitality reduced, as it is very apt to do when, for instance, deprived of the chemical and electrical effect of sun or daylight, as in the night, so that it does not readily separate itself from the dead leucocytes, the dyspnea would return, and the expectorations cease or become greatly diminished. A striking proof that the dyspnea at this time was due to an excessively large number of leucocytes in the blood was found in the fact that it would disappear like magic within twenty or thirty minutes after the administration of a large dose of quinine in solution. During the eliminating and expectorating stage, dyspneic spells of this nature would occasionally suddenly develop coming on nearly as often during daylight as night darkness. When coming on in the daytime the dyspnea would usually appear an hour or so after the ingestion of food, or when the digestion leucocytosis was at its height.

Not infrequently, however, quite severe attacks would come on suddenly, and without any warning, while the patient was up and about, due to an accumulation of leucocytes or mucus in the trachea, just as one's nose will become stuffed up with mucus during an acute coryza, sufficient to exclude the entrance of air. Such attacks would usually last for only twenty minutes or an hour, patient experiencing immediate relief with the first expectoration. There was no regularity about the coming of the dyspnea at this stage, sometimes two or three days or nights elapsing without any dyspnea except on exertion. Sleep, however, was almost impossible owing to the incessant cough and free expectoration. In fact, sound, undisturbed slumber was very apt to terminate in a wretched condition, as twice illustrated by this case. On two nights, several days apart, the patient became so exhausted that he dropped into such a sound slumber that the cough center did not respond to the irritation produced by the filtration and accumulation of leucocytes or mucus in the respiratory tubes; for about four hours the filtrated material thus accumulated in the air tubes. When the patient woke it was with a gasp for breath. After a few moments of this truly breathless condition, enough air managed to reach the lungs to produce a cough which soon forced the mucus from the tubes and, with its expulsion, the dyspnea disappeared.

This eliminating and coughing period lasted for about ten days, when it gradually subsided. The patient now began to show possitive signs of malnutrition. He was rapidly losing in weight, his muscles were flabby and he tired very easily on exertion. The diminished alkalinity of blood and scurvy-like condition now manifested itself by producing spongy gums with extravasations of blood and a rapid accumulation of sordes upon the teeth. In about twenty-four hours the process extended to the larynx and, upon hawking, a rusty colored mucus could be raised. From the larynx the process soon extended to the trachea, bronchi and bronchioles, resulting in a frequent cough, that was accompanied by a feeling of soreness in the chest, and the expectorations of a rusty colored sputum, that not infrequently contained streaks of clear blood. When viewed under the microscope, this sputum was found to contain about an equal number of red blood corpuscles, leucocytes and eosinophiles. The expectoration of mucus of this alarming character lasted about five days, and with its cessation the patient rapidly gained in health and vigor which caused him to feel that his troubles for a time, at least, were nearing a close; and so they were, but the blood gave one more beautiful illustration of its diminished alkalinity and infirmity by the production of a purpura, hemorrhagic-like condition, of a patch of skin the size of one's whole hand, located in the right anterior lower portion of the chest.

The patch contained a copious crop of small hemorrhages, the contents of the bulla soon changed to a serum-like substance, which was accompanied by a destruction of the skin, and an intense burning, itching sensation, that soon left a raw surface, which healed very slowly, leaving a scar-like skin that will ever mark its location. A surprising feature about this case is that the patient was not confined to his room one whole day at a time, which thus served to demonstrate the pathology of asthma, when due to an intestinal toxemic leucocytosis, in this convincing and unmistakable manner.

Osler reports a case of asthma in the Johns Hopkins Hospital *Bulletin*, January, 1901, that has many pathological features in common with the case above described. Osler's case was that of a young man, a Pole, who presented the following features: chill, subnormal temperature, cyanosis, extensive purpura, resembling malignant hemorrhagic smallpox, painful muscles, a leucocytosis of 52,000, eosinophiles, 25 per cent. by a differential count. A point to be emphasised in toxemic asthma is that after the toxic leucocytosis, the dead leucocytes are rapidly given up by the blood and, along with other pathological blood constituents, are soon eliminated through the mucous membranes and expelled from the body in one or all of three ways, viz.: expectoration, diarrhea or vomiting.

The special, or if combined principal route of elimination would be determined by both intrinsic and extrinsic etiological factors. The intrinsic factors would be (1) the excessively large number of dead leucocytes and other foreign matter in the blood, together with (2) the essential vital power that the blood has of separating itself from useless material, combined with (3) the fact that the only outlet or "dumping ground" for the blood is the mucous membranes. The extrinsic factors would be (1) the acquired and (2) the induced. Under the acquired we would have habitually an impaired vitality or chronic catarrhal condition of the mucous membrane of the region involved, which would constitute to the blood the line of least resistance, and therefore most naturally its chief "dumping ground." In the asthmatic we find this state of affairs in the mucous membrane of the respiratory tract. That a weakened, chronic catarrhal, mucous membrane, offers the line of least resistance and is taken advantage of by the blood in the expulsion of dead leucocytes and other foreign substances, has been clinically so numerously, vividly and beautifully illustrated that the only strange thing about it is, that more has not been written on the subject.

We continually meet with, especially in early life, cases of so-called "colds" that rapidly alternate with diarrhea. This feature in the pathology of asthma was most convincingly demonstrated by a case of asthma long under my charge, which stated as briefly as possible, is as follows:

Male, aged 70; had asthma from early life until age 65. Paroxysms not very severe, and frequently enjoyed several years of freedom between attacks. Was, however, never entirely free from bronchitis, that was occasionally accompanied by a severe and uncontrollable cough with free expectoration. From 55 to 64 had asthma continually, to such an extent that during these nine years he never attempted to sleep in bed. At about this time had the remainder of a set of decayed teeth removed. Also, at nearly the same period a severe watery putrid diarrhea set in, soon to be followed by a complete abatement of all asthmatic symptoms. The diarrhea becoming alarming, it was checked and the asthma returned, this alternation between diarrhea and asthma was kept up for about a year, when it was decided to leave the diarrhea to nature, and as a result neither the asthma nor the bronchitis ever returned. The diarrhea, though, continued in a very severe form for about three years. At one time it became so alarming as to threaten life. For the last three years he has had about three liquid stools a day. Has gained in weight, and is possessed of an unusual amount of activity, robustness and endurance, for a man of seventy.

The induced or the process of inducing the route of elimination can in many cases of severe paroxysms be most gratefully utilised. Whether it is best in a given case to induce or stimulate elimination to clear the blood, or to diminish the number of leucocytes by the administration of a large dose of quinine in solution, can only be determined by a vast amount of experience and a thorough knowledge of the case.

A few general rules useful as a guide will here be mentioned: (1) if there has been quite a long interval between attacks and the attack was ushered in with three or four weeks' prodomal symptoms, indicating toxemia such as putrid, decomposing or acid fermenting stools, with slow glandular enlargement, indicated in the respiratory apparatus by tight collars, change of voice, and the like, and the blood analysis denoting eosinophiles, lowered specific gravity, diminished alkalinity and a leucocytosis, it would be best to adopt the slower method and, in fact, we would be compelled to, as quinine here is nearly useless, for it would only affect the leucocytosis. The route of elimination here to be chosen would be the bowels. The eliminating agents preferably to be chosen are calomel and soda, followed by a saline purge and later by potassium iodid, which agent would favor oxygenation by increasing the alkalinity of the blood and diminishing the enlarged respiratory glands; (2) elimination through the mucous membrane of the stomach by vomiting or lavage, can but seldom be advantageously utilised. Such a case would be where the stomach is empty, the intestinal toxemia cured or improved, or where there is a slight nausea already existing; (3) elimination through the mucous membrane of the respiratory apparatus by expectoration usually exists, and seldom, if ever, needs inducement or stimulation; (4) in succeeding paroxysms and after the correction of the above mentioned abnormalities as far as possible, by elimination and the like, the pathology of asthma changes and the only etiological lesion is the leucocytosis, which, when once started, is quite apt to persist for a long time. Here the administration of a large dose of quinine in solution will through its leucocytic action have a magic effect.

Toxins producing the asthmatic leucocytosis do not alone originate in the intestinal canal, as for instance the toxin of syphilis often produces a leucocytosis, eosinophilæ and asthma. The eosinophiles in this case would no doubt come largely from the bone-marrow. The prognosis of the toxic group of asthmas is far more grave than that of the other varieties. When unabated by treatment it usually terminates suddenly, in early adult life, with a cerebral hemorrhage. I can now recall two such cases. When abated by treatment it not infrequently grades off with advancing years into the group now to be considered the anemic variety.

3. *Asthma due to Anemia.*—Cases forming this group usually give a history of malarial infection, intestinal indigestion, syphilis, lead poisoning, or asthmatics, who have had the other varieties of asthma in infancy and adult life respectively. But it matters not what the etiological factors might have been, the pathology of this group is short and uncomplicated, as it lies entirely with the blood's inability to absorb or carry a sufficient amount of oxygen. The blood's inability to perform its oxygenating functions properly in the anemia of the asthmatic, is not alone due to a quantitative deficiency in its hemoglobin as in other anemias, but also to the unfavorable hemoglobin environments. Not infrequently the worst stages of a rapidly fatal pernicious anemia will not so greatly cripple the blood's oxygenating power as will a mild anemia, plus the asthmatic peculiarities. There perhaps are other reasons for not more frequently finding a more marked and distressing dyspnea accompanying our grave anemias. One reason for this would be found in the fact that the great majority of cases of pernicious anemia have a relatively high percentage of hemoglobin, due probably to a degeneration and destruction of the cell substance, leaving the hemoglobin in solution.

Cabot gives the following rule: Cases whose color index is low, and in which the average diameter of the red cells is normal are apt to be gaining at that time, while those with high color index are apt to be losing at that time. This nicely explains how a grave anemia might give but little respiratory discomfort, while a mild anemia, under certain circumstances, could produce an agonising dyspnea. The most prominent and constant pathological factors producing the asthmatic dyspnea, when of the anemic variety, are, (1) a deficiency in the hemoglobin and a diminution in corpuscle substance, as appreciated by lack of coloring matter and a lowering of the specific gravity; (2) a quite constant and appreciable diminution in the blood's alkalinity, said normal alkalinity constituting the basic principle of the blood's oxygenation; (3) causes one and two, complicated with a mild leucocytosis, and a slight impairment of rouleaux formation—except when of malarial origin—due to the toxic plasma and its resultant catarrhal condition of the respiratory tract; (4) a diminution in animal magnetism or electricity. It can be quite positively and satisfactorily proven that there is a decided deficency in animal electro-magnetism during an attack of this variety of asthma.

There is no doubt but that the numerous chemical exchanges of life with all its complexity are largely if not entirely dependent upon electrical forces. Therefore, it would appear as a self-evident fact that in such a delicate chemical change as oxygenation, a diminution in this electro-magnetic force would constitute in the asthmatic a pathological feature. The four above-mentioned pathological facts furnish a very perfect picture of the pathology of the anemic variety, and serve also to most beautifully illustrate upon what a delicate scale its pathology is balanced. This delicate balancing of the pathology offers a rational explanation for its pronounced and rapid oscillation up or down for seemingly trivial causes. The pathological division between attacks and freedom is so frail and sensitive to altitude, atmospheric and telluric surroundings, that attacks are immediately brought on or relieved by their changes.

Thus it is that we have an innumerably long list of exciting factors that will bring on attacks and as long a list of curative measures. There is not much of pathological interest about the sputum or urine of the uncomplicated anemic variety. Curschmann's spirals do not so regularly appear in the sputum of the anemic asthmatic, and their appearance in periodically increasing numbers would indicate a complication with a toxic leucocytosis. The only interesting feature about the urine is the rapidity with which it makes such extreme changes both as regards quantity and quality, thus again portraying the blood's unstableness.

Asthmatics whose dyspnea returns night after night, so that they are unable to sleep in bed for years at a time, belong to this class. The vast majority of cases, though, experience periods of freedom under favorable circumstances. The pathology of the different kinds of asthma as here constructed proves positively, conclusively and satisfactorily, that the lungs and nerves take no part in any of the phenomena of asthma, other than the performance of a physiological duty, which is directly contrary to the anciently established neurotic theory that has so unflinchingly withstood the speculations of all past ages.

